# Nature of β-1,3-Glucan-Exposing Features on Candida albicans Cell Wall and Their Modulation

**DOI:** 10.1128/mbio.02605-22

**Published:** 2022-10-11

**Authors:** Leandro José de Assis, Judith M. Bain, Corin Liddle, Ian Leaves, Christian Hacker, Roberta Peres da Silva, Raif Yuecel, Attila Bebes, David Stead, Delma S. Childers, Arnab Pradhan, Kevin Mackenzie, Katherine Lagree, Daniel E. Larcombe, Qinxi Ma, Gabriela Mol Avelar, Mihai G. Netea, Lars P. Erwig, Aaron P. Mitchell, Gordon D. Brown, Neil A. R. Gow, Alistair J. P. Brown

**Affiliations:** a Medical Research Council Centre for Medical Mycology, University of Exeter, Exeter, United Kingdom; b Aberdeen Fungal Group, Institute of Medical Sciences, University of Aberdeen, Aberdeen, United Kingdom; c Bioimaging Unit, University of Exeter, Exeter, United Kingdom; d Exeter Centre for Cytomics, University of Exeter, Exeter, United Kingdom; e Aberdeen Proteomics Facility, Rowett Institute, University of Aberdeen, Aberdeen, United Kingdom; f Department of Internal Medicine, Radboud University Medical Center, Nijmegen, Netherlands; g Radboud Center for Infectious Diseases, Radboud University Medical Center, Nijmegen, Netherlands; h Department for Immunology & Metabolism, Life and Medical Sciences Institute (LIMES), University of Bonn, Bonn, Germany; i Johnson-Johnson Innovation, EMEA Innovation Centre, London, United Kingdom; j Department of Microbiology, University of Georgia, Athens, Georgia, USA; University of Texas Health Science Center

**Keywords:** *Candida albicans*, cell wall, β-1,3-glucan, protein kinase A, Mig1, Mig2, Sin3, Xog1, Eng1

## Abstract

Candida albicans exists as a commensal of mucosal surfaces and the gastrointestinal tract without causing pathology. However, this fungus is also a common cause of mucosal and systemic infections when antifungal immune defenses become compromised. The activation of antifungal host defenses depends on the recognition of fungal pathogen-associated molecular patterns (PAMPs), such as β-1,3-glucan. In C. albicans, most β-1,3-glucan is present in the inner cell wall, concealed by the outer mannan layer, but some β-1,3-glucan becomes exposed at the cell surface. In response to host signals, such as lactate, C. albicans induces the Xog1 exoglucanase, which shaves exposed β-1,3-glucan from the cell surface, thereby reducing phagocytic recognition. We show here that β-1,3-glucan is exposed at bud scars and punctate foci on the lateral wall of yeast cells, that this exposed β-1,3-glucan is targeted during phagocytic attack, and that lactate-induced masking reduces β-1,3-glucan exposure at bud scars and at punctate foci. β-1,3-Glucan masking depends upon protein kinase A (PKA) signaling. We reveal that inactivating PKA, or its conserved downstream effectors, Sin3 and Mig1/Mig2, affects the amounts of the Xog1 and Eng1 glucanases in the C. albicans secretome and modulates β-1,3-glucan exposure. Furthermore, perturbing PKA, Sin3, or Mig1/Mig2 attenuates the virulence of lactate-exposed C. albicans cells in *Galleria.* Taken together, the data are consistent with the idea that β-1,3-glucan masking contributes to *Candida* pathogenicity.

## INTRODUCTION

Pathogenic fungi impose a major burden upon human health globally (1). For *Candida* infections alone, over one hundred million women suffer recurrent vaginitis infections, and systemic *Candida* infections kill more than 200,000 individuals each year (1). Even with antifungal therapy, mortality rates for bloodstream infections lie between 10 and 20% (2).

The susceptibility of an individual to fungal infection is largely dependent on their immune status (3–5). For example, neutropenic patients are susceptible to systemic candidiasis, aspergillosis, and cryptococcal meningitis, whereas those with defects in T-cell-mediated immunity often suffer mucosal *Candida* infections (1, 5, 6). Immune recognition of colonizing fungal cells is driven by host receptors (pattern recognition receptors [PRRs]), expressed by innate immune cells such as macrophages and neutrophils, which detect the presence of pathogen-associated molecular patterns (PAMPs), some of which lie on the surface of the fungal cells (7–10). The recognition of fungal PAMPs by host PRRs triggers an array of antifungal responses that include phagocytosis, the production of neutrophil extracellular traps, and the release of cytokines and chemokines which recruit immune cells to the infection site and activate adaptive immunity (5, 9–12). The PAMP β-1,3-glucan is recognized by receptors such as the C-type lectin receptor dectin-1 (CLEC6A), the nucleotide-oligomerization domain (NOD)-like receptor NLRP3, and complement receptor 3 (9, 13–15). Of these β-1,3-glucan receptors, dectin-1 plays a dominant role in antifungal immunity, although the recognition of additional PAMPs is required for a full antifungal response (16–22).

Opportunistic fungal pathogens that have evolved as commensals of mammals, such as Candida albicans, have developed strategies to evade antifungal immune defenses (23, 24). For example, C. albicans can actively counteract immune defenses by secreting aspartate proteases and superoxide dismutases (25–27). The fungus can also escape phagocytic killing through hyphal development, active manipulation of phagolysosomal pH, the induction of robust oxidative stress responses, and via metabolic competition for glucose (28–34).

An additional strategy is to avoid immune recognition. For example, low levels of β-1,3-glucan exposure by C. albicans correlate with enhanced colonization of the gastrointestinal tract (35). β-1,3-Glucan is an essential component of the inner layer of the C. albicans cell wall (36, 37) and is normally hidden from immune recognition by the outer layer of mannan fibrils (8, 38–41). This fungus also expresses β-1,3-glucanases (Xog1 and Eng1) that are capable of reducing β-1,3-glucan exposure (42, 43), and C. albicans appears to have evolved mechanisms to induce the expression of these enzymes when a phagocytic attack is imminent (44). The fungus exploits specific host-imposed signals, such as lactate, hypoxia, iron limitation, and ambient pH, to modulate β-1,3-glucan masking and, thereby, influence phagocytic recognition and cytokine induction (43, 45, 46).

Masking of β-1,3-glucan in response to lactate, hypoxia, or iron limitation is activated via different evolutionarily conserved signaling pathways (43, 47, 48). Lactate activates β-1,3-glucan masking through Gpr1, a homologue of the mammalian lactate receptor (49), whereas the hypoxic signal is transduced via mitochondrial reactive oxygen species (47) and iron limitation is transduced via the Ftr1 iron transceptor (48). These signals converge on protein kinase A (PKA), which is essential for β-1,3-glucan masking (47, 48). However, the downstream mechanisms by which PKA regulates β-1,3-glucanase levels to mediate β-1,3-glucan masking remains obscure.

In this study, we explored the nature of β-1,3-glucan exposure in C. albicans, revealing two main types of β-1,3-glucan-exposing features: at septal junctions and bud scars and at punctate foci on the lateral cell wall. Both of these contribute to immune recognition, and both are attenuated by lactate-induced β-1,3-glucan masking. We also tested whether downstream effectors, which are known to mediate the PKA regulation of cellular processes in other fungi (50–59), influence β-1,3-glucan masking in C. albicans. The histone deacetylase Sin3 and the two homologues of Saccharomyces cerevisiae Mig1 in C. albicans (Mig1 and Mig2) (50) were found to modulate β-1,3-glucan exposure. These findings have important implications for immune detection and fungal infection.

## RESULTS

### Regulation of β-1,3-glucan exposure at bud scars and punctate foci.

**FIG 1:**
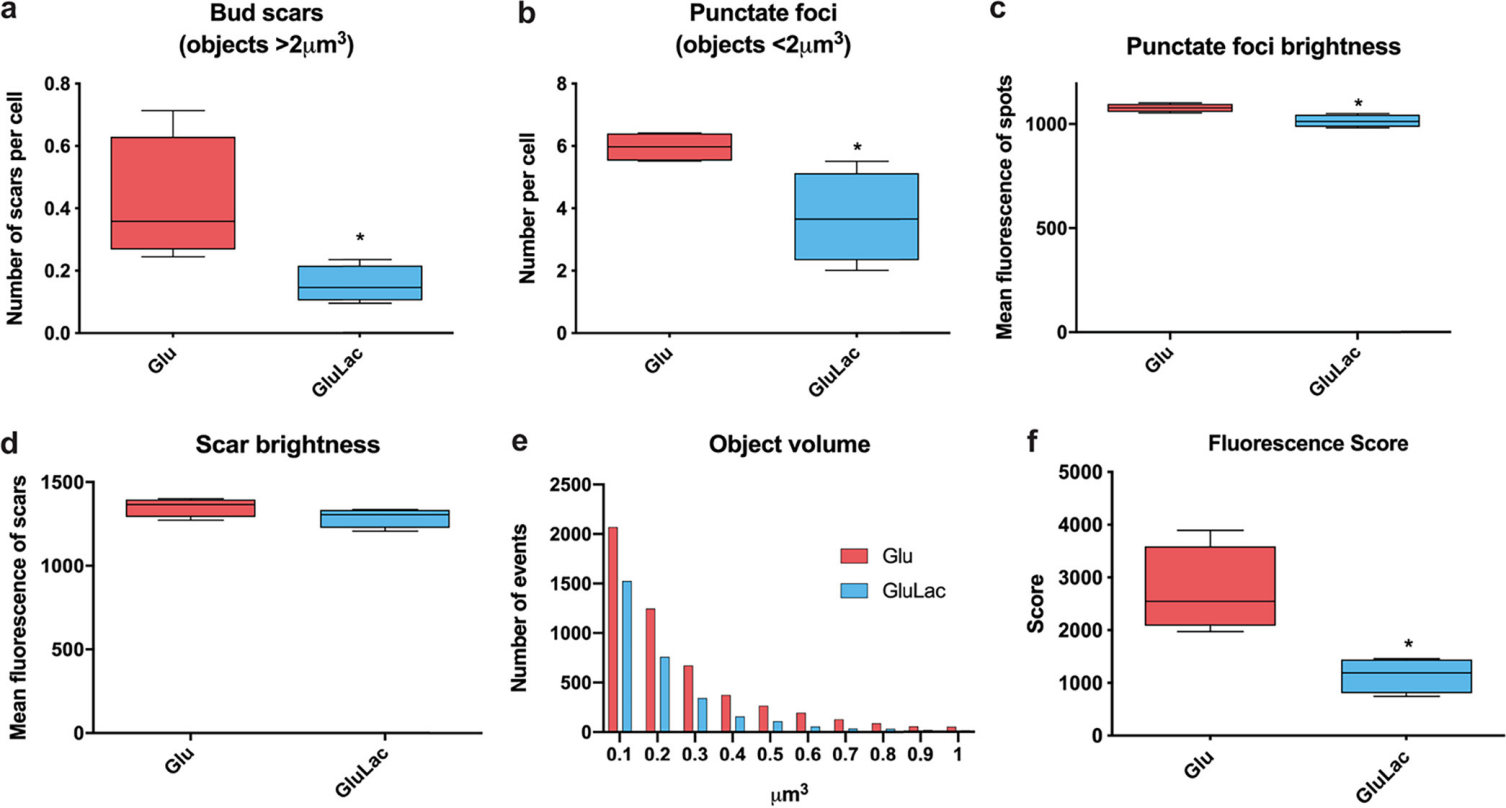
Regulation of β-1,3-glucan exposure at punctate foci and bud scars. Quantitative fluorescence microscopy was used to examine the impact of lactate on the exposure of β-1,3-glucan at punctate foci and bud scars. C. albicans SC5314 cells were grown in GYNB (Glu; red) or GYNB containing lactate (GluLac; blue), fixed, and stained with Fc-dectin-1. These cells were examined using a Nikon Eclipse Ti UltraVIEW VoX spinning-disk microscope, and the volume and intensity of β-1,3-glucan-exposing features were quantified with Volocity software: septal junctions and bud scars, >2 μm^3^; punctate foci, <2 μm^3^. (a) Distribution of β-1,3-glucan-exposing bud scars. (b) Number of β-1,3-glucan-exposing punctate foci showing volumes smaller than 2 μm^3^ per cell. (c) Mean fluorescence intensity of these punctate foci (brightness). (d) Mean fluorescence intensity of these bud scars (brightness). (e) Object volume showing the number of the events observed. (f) Fluorescence scores for cells grown in GYNB or GYNB containing lactate. This fluorescence score combines the number, volume, and brightness of all β-1,3-glucan-exposing features per cell. Means and standard deviations are for data from four independent experiments, in which a total of approximately 850 cells were analyzed for each condition. The data were analyzed using an unpaired *t* test (*, *P* < 0.05).

We measured the effects of lactate-induced masking upon β-1,3-glucan exposure at septal junctions and bud scars and at the punctate foci on the lateral cell wall of C. albicans. This was achieved by fluorescence imaging of Fc-dectin-1-stained C. albicans SC5314 cells (a wild-type [WT] clinical isolate) and quantification of the three-dimensional (3D) images to measure the fluorescence intensity of individual β-1,3-glucan-exposing features. The septal junctions and bud scars were relatively large (>2 μm^3^), whereas punctate foci were relatively small (<2 μm^3^) (Fig. 1a and b). C. albicans cells exposed to lactate displayed significantly fewer punctate foci than the controls, and the fluorescence intensity of these foci showed a significant reduction (Fig. 1b and c). Glucose-grown C. albicans cells that were exposed to lactate also showed fewer β-1,3-glucan-exposing bud scars (Fig. 1a). These bud scars showed only a slight reduction in their fluorescence intensity, which was not statistically significant (Fig. 1d). These changes were reflected in the frequencies of β-1,3-glucan-exposing objects, observed by volume, for cells that were exposed to lactate and those that were not (Fig. 1e) and in the overall fluorescence score for these cells (which combines the number, volume, and fluorescence intensity of all observed features) (Fig. 1f). Therefore, quantitative fluorescence imaging confirmed the lactate-induced β-1,3-glucan masking phenotype observed by flow cytometry (43, 48, 49).

### Macrophages target β-1,3-glucan-exposing features on the C. albicans cell wall.

**FIG 2:**
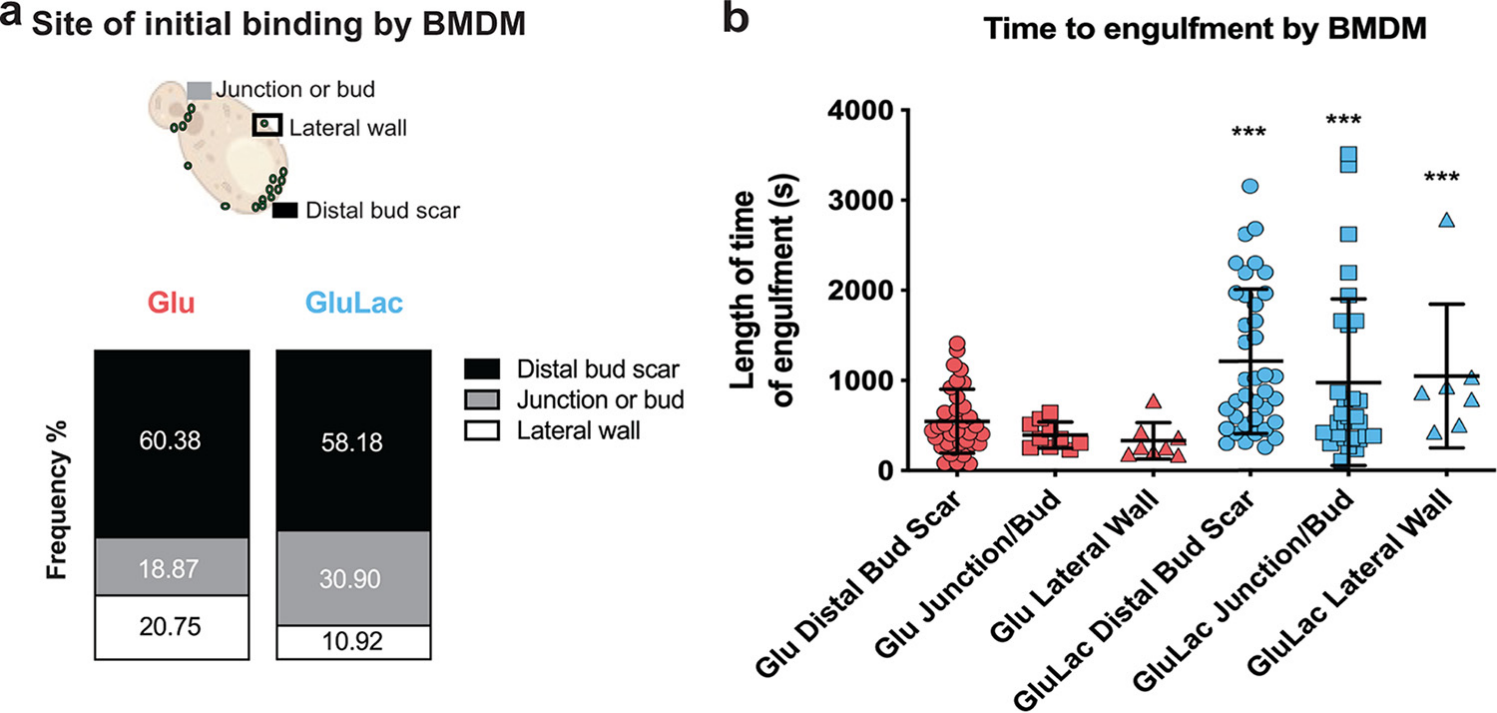
Macrophages target β-1,3-glucan-exposing features on the C. albicans cell wall. C. albicans SC5314 cells were grown in GYNB (Glu; red) or GYNB containing lactate (GluLac; blue), fixed, and washed. These C. albicans cells were then mixed with BMDMs (1:3 macrophage-to-yeast cell ratio), and macrophage-fungal interactions were monitored by live video microscopy at 5-s intervals for 60 min. (a) The site on a target C. albicans cell to which a BMDM first attached was monitored. (b) The time of engulfment from this first attachment to the phagocytosis of the C. albicans cell was also recorded (in seconds). The data are from three independent videos per condition: *n* = 48 events for glucose; *n* = 52 events for glucose plus lactate. Data for the glucose-plus-lactate condition were compared to the corresponding data for the glucose-only control condition using a two-way ANOVA multiple-comparison test (***, *P* < 0.001).

It is well known that β-1,3-glucan is a PAMP that stimulates antifungal immune responses (13, 60–62) and that β-1,3-glucan masking by C. albicans attenuates recognition and cytokine production by innate immune cells (43, 45–49, 63, 64). Therefore, we reasoned that macrophages might exploit β-1,3-glucan-exposing features on the fungal cell wall to recognize and phagocytose C. albicans cells. To test this, we examined interactions between C. albicans cells and murine bone marrow-derived macrophages (BMDMs) by high-speed time-lapse video microscopy (see Videos S1 and S2 in the supplemental material). The C. albicans cells were grown in glucose and either exposed to lactate or not. For these experiments, the cells were not stained with Fc-dectin-1, because this was likely to interfere with β-1,3-glucan–PRR interactions that contribute to fungal recognition. Instead, we monitored the sites at which BMDMs initially bound the C. albicans cells by live imaging with differential interference contrast (DIC). Significantly, the BMDMs bound most frequently to cell wall regions displaying the most β-1,3-glucan exposure (the distal bud scars of mother cells and the septal junctions between mother and daughter cells), and the BMDMs bound less frequently to the lateral cell wall (Fig. 2a).

10.1128/mbio.02605-22.5 VIDEO S1 A macrophage binds its C. albicans cargo at the mother's bud scar. This 120-s video shows the attachment of a BMDM to the bud scar of a mother C. albicans cell, which is then engulfed. Download Movie S1, AVI file, 12.5 MB. Copyright © 2022 de Assis et al. 2022 de Assis et al. https://creativecommons.org/licenses/by/4.0/ This content is distributed under the terms of the Creative Commons Attribution 4.0 International license.

10.1128/mbio.02605-22.6 VIDEO S2 A macrophage binds its C. albicans cargo at the distal end of the mother cell. This 33-s video shows the attachment of a BMDM to the distal end of a mother C. albicans cell (top middle) before engulfment. Download Movie S2, AVI file, 11.1 MB. Copyright © 2022 de Assis et al. 2022 de Assis et al. https://creativecommons.org/licenses/by/4.0/ This content is distributed under the terms of the Creative Commons Attribution 4.0 International license.

We then measured the time it took for a BMDM to completely engulf the target C. albicans cell after it had bound that cell. C. albicans cells that had been exposed to lactate took significantly longer to phagocytose (Fig. 2b). This was the case whether the BMDM had grasped the C. albicans cell by a bud scar, a septal junction, or the lateral cell wall. This was consistent with the concept that the clustering of β-1,3-glucan-binding PRRs promotes efficient phagocytosis of C. albicans (65, 66) and that lactate-induced β-1,3-glucan masking attenuates the responses of innate immune cells to C. albicans.

### Mother cells display more β-glucan exposure.

As S. cerevisiae mother and daughter cells separate during cytokinesis, the septal junction is degraded to release the daughter, leaving a bud scar on the mother (67). This involves the asymmetric synthesis of the Eng1 endoglucanase by the daughter cell, not the mother cell, to degrade β-1,3-glucan in the septum (67, 68). Consequently, S. cerevisiae *eng1* mutants display a cell separation defect (68), and this is also the case for C. albicans (69). Therefore, we compared the levels of β-1,3-glucan exposure on C. albicans mother and daughter cells by flow cytometry.

**FIG 3:**
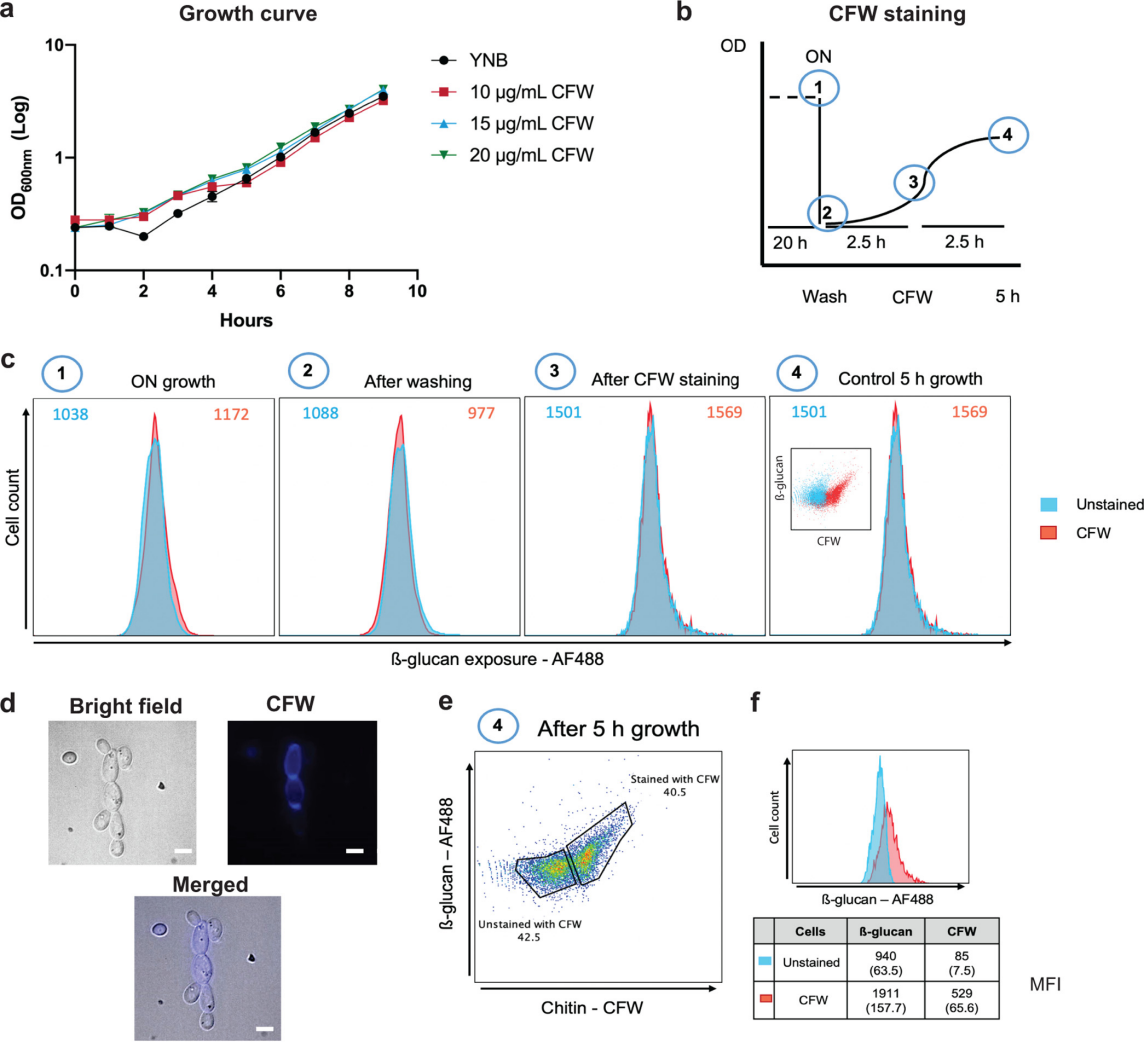
Most glucan exposure is associated with mother cells. Calcofluor white (CFW) staining was used to differentiate older mother cells from younger daughter cells. (a) First, the effect of CFW on growth was tested. C. albicans SN250 cells were grown on GYNB after staining for 5 min with no CFW (yeast nitrogen base [YNB]) or with 10, 15, or 20 μg/mL CFW and washed, and then the growth in medium without CFW was monitored for 9 h. (b) Scheme showing the experimental design. C. albicans SN250 cells were grown overnight in GYNB (1) and then washed and transferred to fresh GYNB at an OD_600_ of 0.2 (2). The cells were then grown for 2.5 h and stained with 20 μg/mL CFW for 5 min (3). The cells were then grown for a further 2.5 h (approximately one doubling time) (4). (c) A control experiment was performed to test whether CFW staining in the context of this experimental design affects subsequent staining with Fc-dectin-1. At each stage (labeled 1 to 4), cultures were split in two: one part was stained for 5 min with 20 μg/mL CFW, whereas the other part was not. Each sample was grown as indicated in GYNB, stained with Fc-dectin-1, and analyzed by flow cytometry. (d) Representative images from microscopy showing C. albicans SN250 wild-type strain presenting yeast and budding yeast forms in bright-field, CFW-stained (mother cells), and merged images (scale bar, 5 μm). (e) After performance of these control experiments, experiments were performed as described for panel c. Then, C. albicans SN250 cells were gated based on their CFW staining, and the levels of β-1,3-glucan exposure were determined for the CFW-positive and CFW-negative subpopulations. (f) The mean CFW MFI and Fc-dectin-1 MFI (with standard deviation) from three independent experiments are shown for each subpopulation.

Before staining with Fc-dectin-1, C. albicans SN250 cells were first stained with calcofluor white (CFW), washed, and then grown in the absence of CFW for a further 2.5 h (approximately one doubling time) to differentiate older cells (mothers; CFW positive) from younger cells (daughters: CFW negative). First, control experiments were performed to confirm that this CFW staining did not interfere significantly with cell growth or β-1,3-glucan staining under our experimental conditions. C. albicans growth was not significantly affected by concentrations of up to 20 μg/mL CFW (Fig. 3a). Also, flow cytometry revealed no significant differences in β-1,3-glucan exposure (median fluorescence intensity [MFI]) between CFW-stained and unstained cells at each stage of this control analysis (Fig. 3b and c).

Having validated the approach, we then compared β-1,3-glucan levels of exposure on mother and daughter cells by flow cytometry. First, the cells were gated into older mother and younger daughter cell populations based on their CFW staining (Fig. 3d). These two cell populations could be clearly differentiated using flow cytometry (Fig. 3e). Then, the intensity of Fc-dectin-1 staining for these two cell populations was determined (MFI) (Fig. 3f). The older mother cells displayed significantly higher levels of β-1,3-glucan exposure than the younger daughter cells (Fig. 3f), which would be consistent with the asymmetric degradation of β-1,3-glucan at septal junctions by daughter cells during cytokinesis in C. albicans, as described for S. cerevisiae (67, 68), to leave residual β-1,3-glucan exposed around the bud scars of mother cells.

### High-resolution microscopy of β-1,3-glucan exposure on yeast cells.

**FIG 4:**
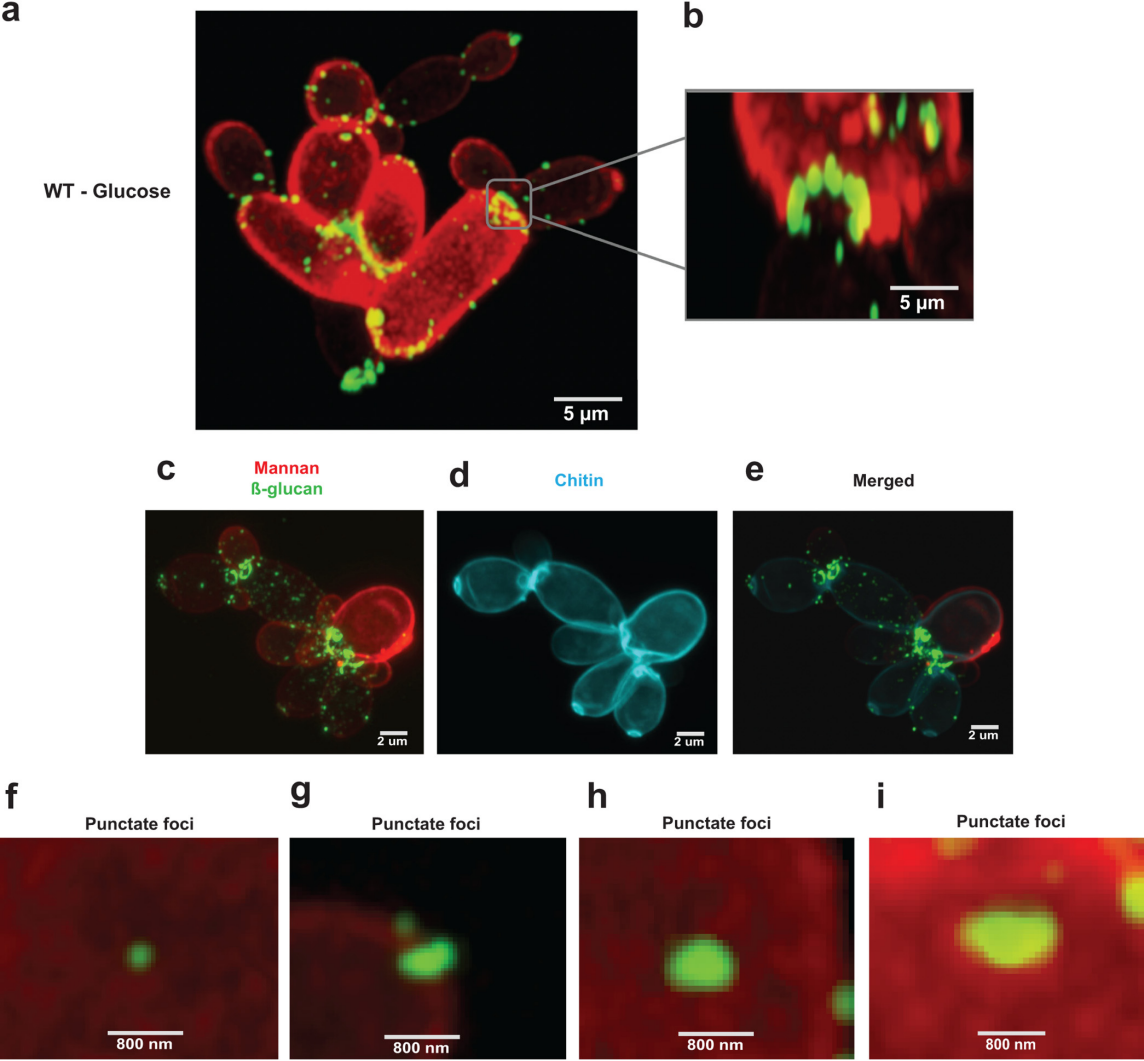
Nature of the β-1,3-glucan exposure on budding cells. (a) C. albicans wild-type (SN250) cells were grown on glucose alone (GYNB) for 5 h and stained with Fc-dectin-1/IgG secondary antibody-AF488 (for β-1,3-glucan exposure) and ConA-AF647 (mannan), and they were examined by high-resolution confocal microscopy (scale bar, 5 μm). (b) Close-up of β-ring structure (septal junctions/bud scars) at a septal junction (scale bar, 5 μm). (c to e) Triple staining for mannan (red), chitin (cyan), and β-glucan (green) showing exposed β-glucan features with overlapped images of stained chitin, thereby highlighting the localization of β-glucan on the bud scar. (f to i) Images of punctate foci of different sizes (scale bar, 800 nm). Panels a to i are representative images from three independent experiments; at least nine images were generated for each condition.

The architecture of the C. albicans cell wall has been studied for many years, culminating in the recent description of a detailed scalar model (39). The outer mannan layer of the cell wall is known to mask most of the β-1,3-glucan, which is present in the inner cell wall (39–41). Nevertheless, some β-1,3-glucan becomes exposed at septal junctions and bud scars and in punctate features on the surface of the lateral cell wall of yeast and hyphal cells (45, 47, 48, 70, 71). However, little is known about the spatial organization of this β-1,3-glucan exposure. Therefore, we characterized β-1,3-glucan-exposing features on the C. albicans cell wall surface by high-resolution confocal microscopy of glucose-grown cells stained with Fc-dectin-1 (for β-1,3-glucan exposure) and concanavalin A (ConA)-AF647 (for mannan) (Fig. 4a; Video S3). The rendered Z-stacks were then used to generate a 3D model using Zeiss Zen (blue edition) software. This revealed that the exposed β-1,3-glucan associated with septal junctions is organized in necklace-like structures with a diameter of approximately 2 μm located close to the mother-daughter neck of dividing cells (Fig. 4b). Triple staining with mannan, chitin, and β-glucan confirmed that these necklace-like structures of exposed β-1,3-glucan were located at mother-daughter junctions and at bud scars (Fig. 4c to e). Meanwhile, the punctate foci, which varied in size from about 0.1 to 1 μm, appeared to traverse the outer mannan layer of the lateral cell wall (Fig. 1e and 4f to i).

10.1128/mbio.02605-22.7 VIDEO S3 Airyscan high-resolution confocal microscopy of β-glucan-exposing features on C. albicans cells. Wild-type (SN250) cells grown in glucose expose β-1,3-glucan at septal junctions/bud scars and at punctate foci on the lateral cell wall (Fig. 7a). High-resolution confocal microscopy and 3D modeling generated using Z-stack assemblies revealed these structures. Exposed β-1,3-glucan is shown in green, and exposed mannan is shown in red. Download Movie S3, AVI file, 2.6 MB. Copyright © 2022 de Assis et al. 2022 de Assis et al. https://creativecommons.org/licenses/by/4.0/ This content is distributed under the terms of the Creative Commons Attribution 4.0 International license.

### Downstream targets of PKA control β-glucan masking.

The masking of β-1,3-glucan in C. albicans is regulated by PKA (48). However, the downstream targets of the PKA pathway that mediate this phenotype are not known. In Aspergillus nidulans, PKA indirectly regulates the transcription factor CreA (the homologue of C. albicans Mig1) via the protein kinase Stk22 (the homologue of C. albicans Sak1) (52). C. albicans contains two Mig1-like genes, *MIG1* and *MIG*2, which display some functional redundancy (50). In the presence of glucose, A. nidulans CreA accumulates in the nucleus and recruits the histone deacetylase Sin3 to mediate catabolite repression (53). In S. cerevisiae, Sin3 interacts with the corepressors Tup1 and Cyc8/Ssn6 (54), which mediate transcriptional repression by Mig1. Significantly, the targets of PKA phosphorylation in C. albicans include Sin3 (51). Bringing these observations together, we reasoned that Sin3 and Mig1/2 may contribute to PKA-mediated regulation.

We tested whether Sin3 physically interacts with Mig1 or Mig2 by identifying subsets of C. albicans proteins that coimmunoprecipitate with Sin3-FLAG_2_. As expected, we reproducibly identified orthologues of other components of the histone deacetylase complex in S. cerevisiae, such as Rpd3 (e.g., CaRpd3, CaRpd31) (Table S2). However, neither Mig1 nor Mig2 was identified.

10.1128/mbio.02605-22.2 TABLE S2 Sin3-FLAG_2_ interacting proteins in Candida albicans cells grown on glucose in the presence or absence of lactate. Download Table S2, XLSX file, 0.5 MB. Copyright © 2022 de Assis et al. 2022 de Assis et al. https://creativecommons.org/licenses/by/4.0/ This content is distributed under the terms of the Creative Commons Attribution 4.0 International license.

**FIG 5:**
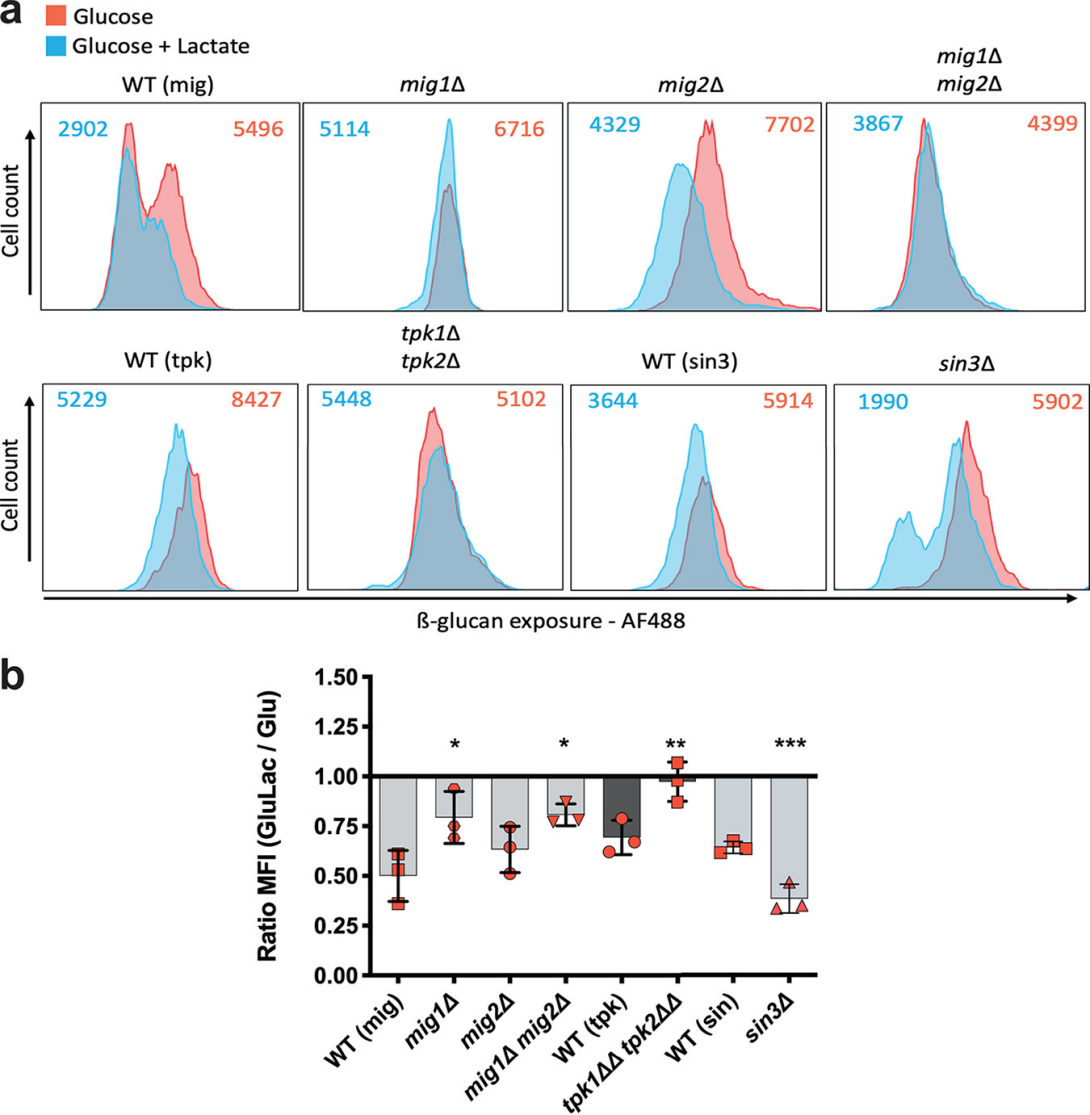
Downstream targets of PKA control β-glucan masking. (a) Flow cytometry of the following C. albicans strains grown for 5 h in GYNB (red) or GYNB containing lactate (blue) and stained with Fc-dectin-1: WT (mig) (SN250), *mig1*Δ, *mig2*Δ, *mig1*Δ *mig2*Δ, WT (tpk) (SN152HLA), *tpk1*Δ *tpk2*Δ, WT (sin3), and *sin3*Δ strains. MFIs for cells grown under each condition are shown at the top of each panel. These profiles represent those obtained in three independent experiments. (b) Fold changes in β-1,3-glucan exposure, calculated as a ratio of the MFI for GluLac to the MFI for Glu. Means and standard deviations of results from three independent experiments are shown. The data were analyzed using two-way ANOVA with Tukey’s multiple-comparison test (*, *P* < 0.05; **, *P* < 0.01; ***, *P* < 0.001).

We then tested the influence of Mig1/2 and Sin3 on lactate-induced β-1,3-glucan masking in C. albicans. Wild-type SC5314 cells were grown in minimal medium containing glucose, exposed to either 0 or 2% lactate for 5 h, and then stained with Fc-dectin-1. The amount of Fc-dectin-1 binding, which reflects β-1,3-glucan exposure levels, was quantified by flow cytometry (Fig. 5a). The impact of lactate on β-1,3-glucan exposure was then calculated by determining the fold change in the MFI in response to lactate (Fig. 5b). Wild-type C. albicans cells consistently displayed lactate-induced β-1,3-glucan masking, and *tpk1*Δ *tpk2*Δ cells lost this phenotype, as described previously (48, 49). Interestingly, cells lacking Mig1 displayed significantly impaired β-1,3-glucan masking, whereas *mig2*Δ cells did not (Fig. 5b). As described previously, Mig1 and Mig2 display partial functional redundancy (50), and our data suggest that Mig1, not Mig2, contributes to the β-1,3-glucan masking phenotype. The C. albicans *sin3*Δ mutant displayed the phenotype opposite to that of the *mig1* mutant: *sin3* cells displayed relatively strong β-1,3-glucan masking compared to their isogenic wild-type control (Fig. 5b). Hence, β-1,3-glucan masking is upregulated by PKA and Mig1 and downregulated by Sin3.

### Expression of genes encoding β-1,3-glucan shaving enzymes is modulated by PKA, Mig1/2, and Sin3.

Previously, we reported that the major secreted exoglucanase, Xog1, shaves β-1,3-glucan from the C. albicans cell surface (42). We also showed that the levels of the secreted endoglucanase, Eng1, are regulated in response to lactate (42), and this endoglucanase has also been shown to contribute to β-1,3-glucan masking in C. albicans (42). We first monitored the expression of genes encoding shaving enzymes by reverse transcriptase quantitative PCR (qRT-PCR). We investigated the impact of PKA, Sin3, and Mig1/2 on *XOG1* and *ENG1* transcript levels during growth on glucose or glucose plus lactate. Previous work showed that β-1,3-glucan exposure is reduced in stationary phase (72), and therefore, stationary- and exponential-phase cells were included in our analyses.

**FIG 6:**
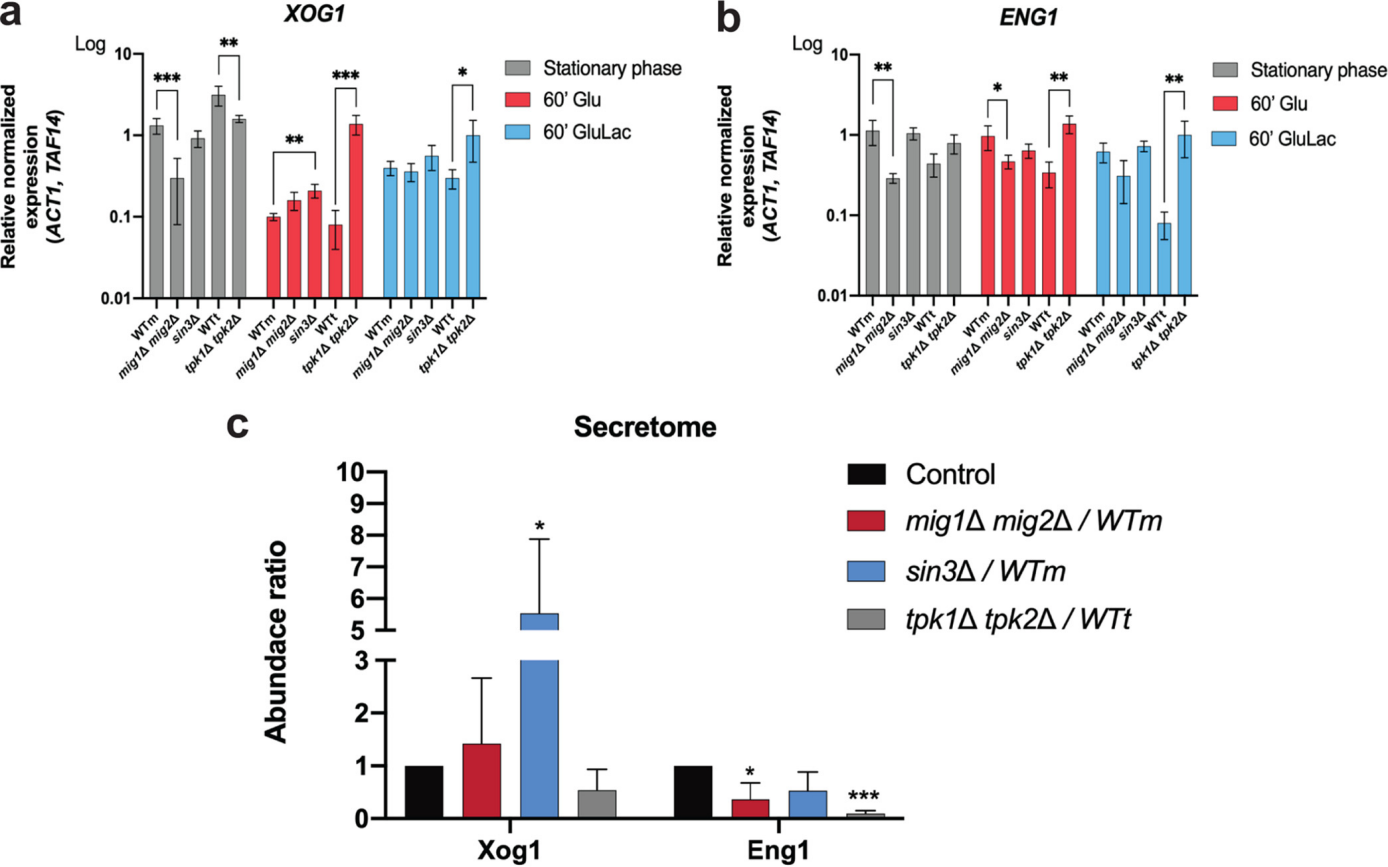
Expression of genes encoding β-1,3-glucan shaving enzymes is modulated by PKA, Mig1/2, and Sin3. (a and b) *XOG1* (a) and *ENG1* (b) transcript levels measured relative to the *ACT1* and *TAF14* internal controls. The following C. albicans strains were grown overnight to stationary phase on GYNB (gray) or for 60 min on GYNB (Glu; red) or GYNB containing lactate (GluLac; blue): WTm (SN250), *mig1*Δ *mig2*Δ mutant, *sin3*Δ mutant, WTt (SN152HLA), and *tpk1*Δ *tpk2*Δ mutant. (c) Relative levels of Xog1 and Eng1 in the C. albicans secretome as quantified by LC-MS/MS. Cells were grown in GYNB plus lactate for 5 h. The level of the target protein in the mutant secretome is divided by the level in the secretome of the corresponding isogenic parental strain; strains include WTm (SN250), *mig1*Δ *mig2*Δ mutant, *sin3*Δ mutant, WTt (SN152HLA), and *tpk1*Δ *tpk2*Δ mutant. The data, which represent means of results from three replicate experiments, were analyzed using two-way ANOVA with Tukey’s multiple-comparison test (*, *P* < 0.05; **, *P* < 0.01; ***, *P* < 0.001).

In stationary-phase cells, *XOG1* and *ENG1* transcript levels were decreased following Mig1/2 inactivation, suggesting that Mig1 and Mig2 are required for normal levels of expression under these conditions (Fig. 6a and b). However, these effects were less dramatic for exponentially growing cells, whether or not they were exposed to lactate. Sin3 inactivation did not affect *XOG1* and *ENG1* transcript levels dramatically, but the loss of PKA (*tpk1*Δ *tpk2*Δ) reduced *XOG1* transcript levels in stationary-phase cells and increased *XOG1* and *ENG1* levels in exponential-phase cells, whether or not lactate was present (Fig. 6a and b).

These changes in the levels of β-1,3-glucanase-encoding transcripts did not correlate well with the impact of PKA, Sin3, and Mig1/2 upon β-1,3-glucan masking (Fig. 5b). For example, the inactivation of PKA led to elevated *XOG1* mRNA levels (Fig. 6a) but reduced lactate-induced β-1,3-glucan masking (Fig. 5b). Therefore, we compared the secretomes of *mig1*Δ *mig2*Δ, *sin3*Δ, and *tpk1*Δ *tpk2*Δ cells to those of their isogenic wild-type controls during lactate exposure, when masking is activated. The levels of secreted Xog1 were dramatically reduced in *tpk1*Δ *tpk2*Δ cells and elevated in *sin3*Δ cells (Fig. 6c; Table S1), which was consistent with the effects of these mutations upon β-1,3-glucan masking (Fig. 5b). The levels of secreted Eng1 were reduced in *mig1*Δ *mig2*Δ cells (Fig. 6c), which might contribute to their attenuated β-1,3-glucan masking (Fig. 5b). Therefore, the levels of Xog1 and Eng1 in the secretome correlated with their roles in β-1,3-glucan masking, but their transcript levels did not (Fig. 6a and b). Therefore, the levels of Xog1 and Eng1 in the secretome appear to be regulated posttranscriptionally.

10.1128/mbio.02605-22.1 TABLE S1 Secretomes of Candida albicans strains grown on glucose in the presence or absence of lactate. WTm (SN250) was the parental control for the *mig1*Δ *mig2*Δ and *sin3*Δ strains, and WTt (SN152HLA) was the control for the *tpk1*Δ *tpk2*Δ strain. Download Table S1, XLSX file, 0.8 MB. Copyright © 2022 de Assis et al. 2022 de Assis et al. https://creativecommons.org/licenses/by/4.0/ This content is distributed under the terms of the Creative Commons Attribution 4.0 International license.

### Impact of PKA, Sin3, and Mig1/2 upon β-1,3-glucan-exposing features on the cell wall.

**FIG 7:**
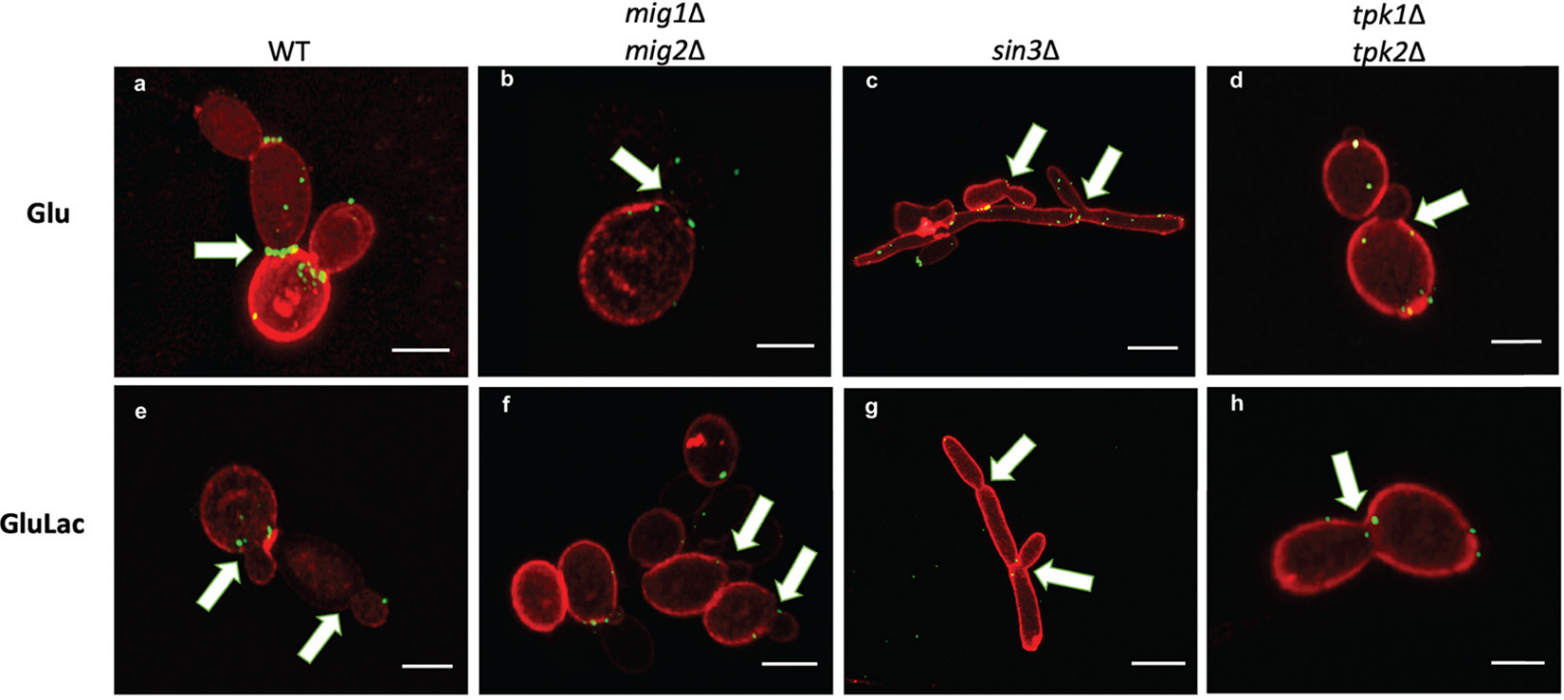
Impact of PKA, Sin3, and Mig1/2 upon β-1,3-glucan exposure at septal junctions/bud scars and punctate foci. The effects of inactivating PKA, Sin3, or Mig1/2 upon β-1,3-glucan-exposing features was examined by confocal high-resolution fluorescence imaging using Airyscan. C. albicans cells were grown on for 5 h on GYNB (Glu) or GYNB containing lactate (GluLac), fixed, and double-stained with Fc-dectin-1 (β-1,3-glucan exposure; green) and ConA (mannan; red). (a and e) WT (SN250); (b and f) *mig1*Δ *mig2*Δ mutant; (c and g) *sin3*Δ mutant; (d and h) *tpk1*Δ *tpk2*Δ mutant. The scale bar represents 5 μm; white arrows indicate septal junctions. These are representative images from three independent experiments with the acquisition of at least 9 images for each condition.

We then examined the influence of PKA, Sin3, and Mig1/2 on the nature of β-1,3-glucan exposure on C. albicans cells by confocal high-resolution fluorescence microscopy after growth in medium supplemented with glucose or glucose plus lactate (Fig. 7). As observed previously, β-1,3-glucan was exposed in β-ring necklace-like structures on glucose-grown wild-type cells (Fig. 4b), but when these cells were exposed to lactate, they lacked this β-1,3-glucan structure (Fig. 7a and e). Interestingly, irrespective of whether lactate was present, the *mig1*Δ *mig2*Δ, *sin3*Δ, and *tpk1*Δ *tpk2*Δ mutants all displayed low levels of β-1,3-glucan exposure at their septal junctions in comparison with those of control wild-type C. albicans cells grown on glucose alone (Fig. 7, arrows; Videos S4 to S6). The presence of lactate reduced the growth of wild-type and *mig1/2*Δ cells but not of the *sin3*Δ and *tpk1/2*Δ mutants (Fig. S1a). Also, the *sin3*Δ mutant formed filaments under all of the conditions tested (Fig. 7c and g). These observations were consistent with the idea that growth (72) and filamentation influence β-1,3-glucan exposure. Additionally, the *tpk1*Δ *tpk2*Δ, *sin3*Δ, and *mig1*Δ *mig2*Δ mutations affected the resistance of C. albicans to the cell wall stressors calcofluor white and Congo red (Fig. S1b) which, together with the changes in β-1,3-glucan exposure, was consistent with the view that these mutations affect cell wall functionality (50, 51, 73).

10.1128/mbio.02605-22.8 VIDEO S4 Airyscan high-resolution confocal microscopy of exposed β-glucan on *mig1*Δ *mig2*Δ cells. C. albicans *mig1*Δ *mig2*Δ cells were grown in glucose plus lactate and subjected to high-resolution fluorescence microscopy and 3D modelling. These *mig1*Δ *mig2*Δ cells show reduced β-1,3-glucan exposure at septal junctions and bud scars and reduced mannan staining. Exposed β-glucan is shown in green, and exposed mannan is shown in red. Download Movie S4, AVI file, 2.2 MB. Copyright © 2022 de Assis et al. 2022 de Assis et al. https://creativecommons.org/licenses/by/4.0/ This content is distributed under the terms of the Creative Commons Attribution 4.0 International license.

10.1128/mbio.02605-22.9 VIDEO S5 Airyscan high-resolution confocal microscopy of exposed β-1,3-glucan on *sin3*Δ cells. C. albicans *sin3*Δ cells grown in glucose plus lactate form filaments and show limited β-1,3-glucan exposure at septal junctions and bud scars and reduced mannan staining. Exposed β-glucan is shown in green, and exposed mannan is shown in red. Download Movie S5, AVI file, 1.6 MB. Copyright © 2022 de Assis et al. 2022 de Assis et al. https://creativecommons.org/licenses/by/4.0/ This content is distributed under the terms of the Creative Commons Attribution 4.0 International license.

10.1128/mbio.02605-22.10 VIDEO S6 Airyscan high-resolution confocal microscopy of exposed β-1,3-glucan on *tpk1*Δ *tpk2*Δ cells. C. albicans *tpk1*Δ *tpk2*Δ cells grown in glucose plus lactate show minimal β-1,3-glucan exposure at septal junctions and bud scars but do expose β-1,3-glucan at punctate foci. Exposed β-glucan is shown in green, and exposed mannan is shown in red. Download Movie S6, AVI file, 1.8 MB. Copyright © 2022 de Assis et al. 2022 de Assis et al. https://creativecommons.org/licenses/by/4.0/ This content is distributed under the terms of the Creative Commons Attribution 4.0 International license.

10.1128/mbio.02605-22.4 FIG S1 Glucose-grown and glucose-lactate-grown cells after 5 h. Strains were inoculated in GYNB medium and grown for 16 h at 30°C. The next day, cells were washed twice with water, inoculated at 0.2 OD/mL, and allowed to grow for 5 h. (a) The glucose and glucose-lactate-grown cells were quantified by OD_600nm_. (b) Strains were grown in complete medium for 16 h at 30°C and then washed twice and diluted to 1.0 OD/mL before dropout spot inoculation was done. A serial dilution was applied, namely, 1.0, 0.1, 0.001, 0.001, and 0.0001 OD/mL, and these dilutions were inoculated using a 96-well replica plater. The plates were grown for 3 days at 30°C. The cell wall stress agents used, CFW (calcofluor white) and CR (Congo red), are shown in increased concentrations, represented as micrograms per milliliter. The data are the average of three independent experiments, and the replicates are presented as black circles (glucose) and black squares (glucose plus lactate). Standard deviations represent the averages of results from three biological replicates. *, *P* < 0.05; **, *P* < 0.01; ***, *P* < 0.001; two-way ANOVA multiple-comparison test using the WT (isogenic background) as a reference for each condition. Download FIG S1, PDF file, 1.5 MB. Copyright © 2022 de Assis et al. 2022 de Assis et al. https://creativecommons.org/licenses/by/4.0/ This content is distributed under the terms of the Creative Commons Attribution 4.0 International license.

### β-1,3-Glucan exposure on yeast and filamentous C. albicans cells.

Previously, we focused primarily on lactate-induced β-1,3-glucan masking during growth of the yeast form of C. albicans (43, 74). However, the levels of β-1,3-glucan exposure change during growth and during yeast-hypha morphogenesis (72). Therefore, we used imaging flow cytometry to measure β-1,3-glucan exposure on yeast and filamentous cells in wild-type and mutant cell populations.

**FIG 8:**
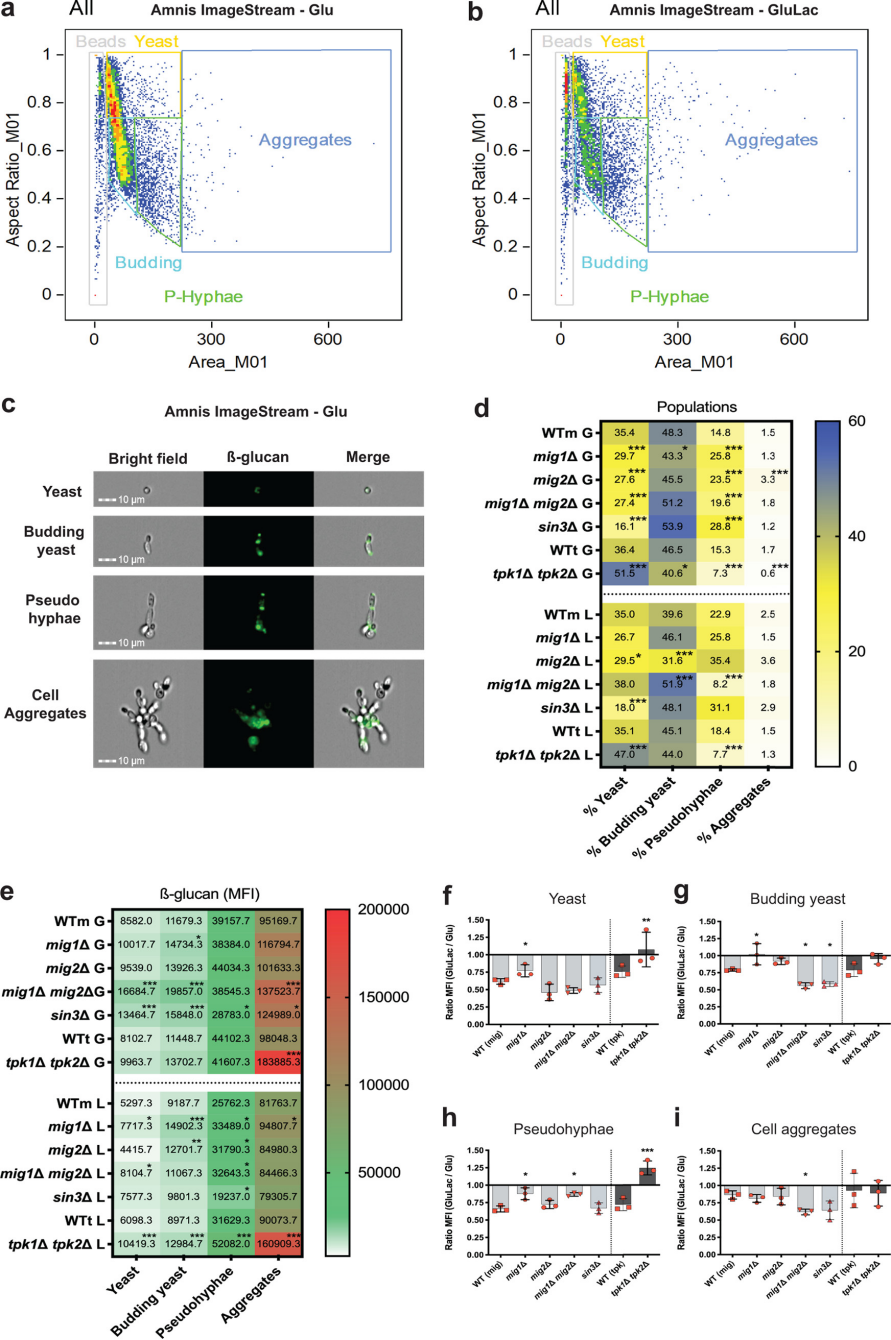
Regulation of β-1,3-glucan exposure in yeast and pseudohyphal cells. C. albicans cells were grown for 5 h in GYNB or GYNB containing lactate and stained with Fc-dectin-1. The proportions of yeast and pseudohyphal cells in these populations, and their levels of β-1,3-glucan exposure, were then analyzed by imaging flow cytometry. Based on cell morphology observed by imaging, and the distribution of the cells by cytometry, cell populations were gated into unbudded yeast cells, budding yeast, pseudohyphae, and filamentous cells. (a) Gating of cells grown on GYNB (glucose alone). (b) Gating of cells grown on GYNB containing lactate. (c) Representative images of C. albicans SN250 cells in these subpopulations, including bright-field, fluorescent micrograph, and merged images. (d) Percentages of unbudded yeast cells, budding yeast cells, pseudohyphae, and filamentous cells observed in different C. albicans strains grown for 5 h on GYNB (G) or GYNB containing lactate (L), including WTm (SN250), *mig1*Δ, *mig2*Δ, *mig1*Δ *mig2*Δ, *sin3*Δ, WTt (SN152HLA), and *tpk1*Δ *tpk2*Δ strains. (e) Levels of β-1,3-glucan exposure (MFI) displayed by each subpopulation shown in panel d. (f to i) Fold change in β-1,3-glucan exposure for each subpopulation shown in panel e, calculated as the ratio of GluLac MFI to Glu MFI. Means and standard deviations of results from three independent experiments are shown. The data in panels d to f represent means of results from three independent experiments and were analyzed using two-way ANOVA with Tukey’s multiple-comparison test (*, *P* < 0.05; **, *P* < 0.01; ***, *P* < 0.001).

First, C. albicans wild-type cells were grown on minimal medium containing glucose or glucose plus lactate, stained with Fc-dectin-1, and gated for unbudded yeast cells, budding yeasts, pseudohyphae, and cell aggregates (Fig. 8a to c). There was a predominance of yeast cells under both growth conditions, but the proportion of pseudohyphal cells increased in the presence of lactate (Fig. 8d). The images suggest that pseudohyphae and cell aggregates display higher levels of β-1,3-glucan exposure than unbudded yeast cells (Fig. 8c). This impression was confirmed by determining the MFIs for these cell populations (Fig. 8e). The data also revealed that exposure to lactate modulated the β-1,3-glucan exposure levels to differing extents, depending on the stage of development. For example, yeast cells (budded and unbudded) and pseudohyphae displayed stronger lactate-induced masking than cell aggregates (Fig. 8f to i). However, the cell aggregates represented a small fraction of the total population (Fig. 8d).

Next, we tested the influence of PKA, Sin3, and Mig1/2 upon β-1,3-glucan exposure levels for these cell types by using imaging flow cytometry. Wild-type, *mig1*Δ, *mig2*Δ, *mig1*Δ *mig2*Δ, *sin3*Δ, and *tpk1*Δ *tpk2*Δ cells were grown in glucose-containing minimal medium, with or without lactate, and stained with Fc-dectin-1. Once again, these populations were gated for unbudded yeast cells, budding yeasts, pseudohyphae, and cell aggregates, and the β-1,3-glucan staining on each subpopulation was quantified (MFI). The *tpk1*Δ *tpk2*Δ mutant formed fewer pseudohyphae and cell aggregates, as expected (73, 75), and displayed attenuated lactate-induced β-1,3-glucan masking (Fig. 8f to i), as predicted (47, 48). Sin3 inactivation enhanced β-1,3-glucan masking in budding yeast cells (Fig. 8g), as described before (Fig. 5b). This increased masking also correlated with increased pseudohypha formation by *sin3*Δ cells (Fig. 8d). Meanwhile, *mig1*Δ *mig2*Δ cells displayed decreased pseudohypha formation in the presence of lactate but enhanced β-1,3-glucan masking in budding yeasts (Fig. 8d and e). These observations are consistent with the idea that the *mig1*Δ *mig2*Δ, *sin3*Δ, and *tpk1*Δ *tpk2*Δ mutations influence the β-1,3-glucan exposure of C. albicans cell populations partly through their effects upon growth and development.

### Impact of PKA, Sin3, and Mig1/2 upon C. albicans virulence.

Previously, we showed that lactate attenuates anti-*Candida* immune responses in murine and human cells and enhances the virulence of C. albicans in a mouse model of systemic candidiasis (76, 77). We also showed that a β-glucanase inhibitor attenuates the virulence of C. albicans (43). PKA, Sin3, and Mig1/2 influence β-1,3-glucan masking (Fig. 5b), and therefore, we tested whether inactivating these regulators affects the impact of lactate on C. albicans virulence. To achieve this, we compared the virulence of C. albicans *mig1*Δ *mig2*Δ, *sin3*Δ, and *tpk1*Δ *tpk2*Δ cells after pregrowth on glucose or glucose plus lactate in the Galleria mellonella model of systemic candidiasis (78, 79).

**FIG 9:**
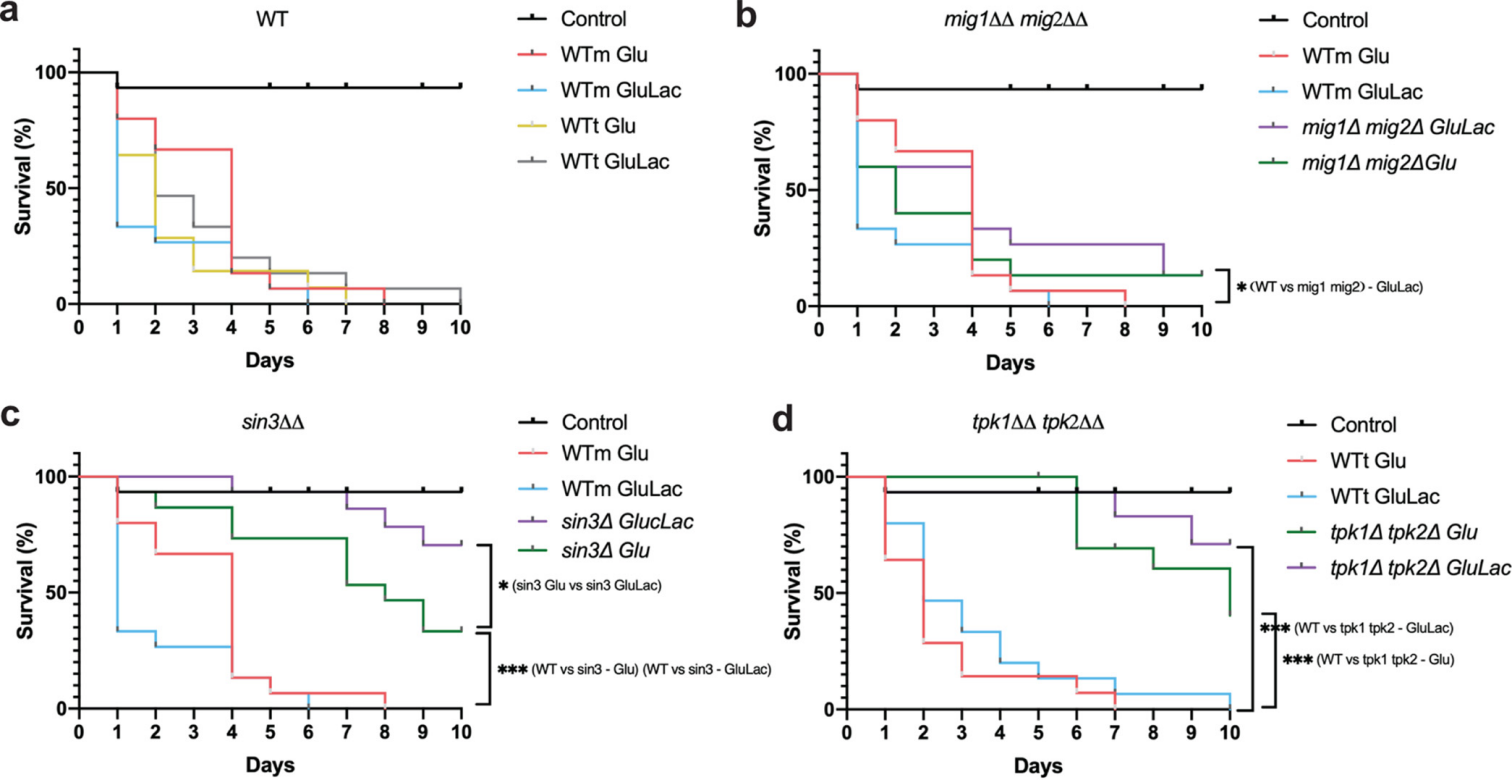
PKA, Sin3, and Mig1/2 contribute to the lactate-enhanced virulence of C. albicans. C. albicans strains were grown either on GYNB (Glu) or GYNB containing lactate (GluLac), and then their virulence was examined using the *Galleria* model. C. albicans cells were harvested, washed, and resuspended at 5 × 10^6^ CFU/mL in PBS, and then 50-μL volumes of these cell suspensions were used to inject the larvae. Control larvae received PBS alone. The survival of the larvae was monitored for 10 days at 37°C in the dark, death and melanin formation were observed daily, and dead insects were removed. Representative graphs from three independent experiments using 15 larvae for each condition are shown. (a) Wild-type strains WTm (SN250) and WTt (SN152HLA): WTm Glu, red; WTm GluLac, blue; WTt Glu, yellow; WTt GluLac, gray. (b) Wild-type WTm (SN250) and *mig1*Δ *mig2*Δ mutant: WTm Glu, red; WTm GluLac, blue; mutant Glu, green; mutant GluLac, purple. (c) Wild-type WTm (SN250) and *sin3*Δ mutant: WTm Glu, red; WTm GluLac, blue; mutant Glu, green; mutant GluLac, purple. (d) Wild-type WTt (SN152HLA) and *tpk1*Δ *tpk2*Δ mutant: WTt Glu, red; WTt GluLac, blue; mutant Glu, green; mutant GluLac, purple. Comparisons of survival curves were done using the log rank (Mantel-Cox) test, with comparison of mutants against their respective parental strains: i.e., WTm (SN250) against *mig1*Δ *mig2*Δ and *sin3*Δ mutants and WTt (SN152HLA) against *tpk1*Δ *tpk2*Δ mutants (*, *P* < 0.05; **, *P* < 0.01; ***, *P* < 0.001).

Unexpectedly, lactate exposure did not enhance the virulence of wild-type C. albicans cells in the *Galleria* model (Fig. 9a). Two related wild-type strains were compared: C. albicans SN250 (WTm) and SN152HLA (WTt). No significant differences in larval survival were observed for fungal cells that were preadapted on glucose plus lactate and their glucose controls. The *tpk1*Δ *tpk2*Δ, *sin3*Δ, and *mig1*Δ *mig2*Δ mutants were then compared to their relevant wild-type controls (Fig. 9b to d). All three mutants were significantly less virulent than their wild-type controls when pregrown on glucose plus lactate. Furthermore, an interesting trend was observed in that all three mutants displayed lower rates of larval killing when they were preadapted in the presence of lactate compared to the corresponding glucose-only controls, although this difference was statistically significant only for the *sin3*Δ cells (Fig. 9b to d). These data are consistent with the idea that Tpk1/2, Sin3, and Mig1/2 contribute to the virulence of C. albicans when cells have been exposed to lactate. β-1,3-Glucan masking could contribute to this enhanced virulence. However, *sin3*Δ cells display lactate-induced β-1,3-glucan masking (Fig. 5b), and yet their virulence was reduced following lactate exposure (Fig. 9c).

## DISCUSSION

C. albicans virulence is a multifactorial phenotype, involving the immune status and the fitness of the host, the presence of a competitive microbiota, and characteristics directly attributable to the fungus (80–83). A combination of fungal virulence factors and fitness attributes promotes the severity of the disease, and these include the secretion of host degradative hydrolases and the pore-forming toxin candidalysin as well as yeast-hypha morphological transitions and robust adaptative responses to local environmental challenges. Following recognition of colonizing fungal cells, the host responds by activating local antifungal responses designed to limit fungal growth and clear the invading fungus (5, 9, 10, 84, 85). However, C. albicans has evolved responses to these host-imposed challenges, one of which is the masking of β-1,3-glucan at its cell surface, which attenuates innate antifungal immune responses (43, 47–49).

Most β-1,3-glucan in the C. albicans cell wall is buried beneath the outer layer of mannan fibrils (38, 39, 41). How then does some β-1,3-glucan become exposed at the cell surface? Our data confirm that β-1,3-glucan becomes exposed in two main types of features: (i) larger features at septal junctions and bud scars (Fig. 4b) and (ii) smaller punctate foci on the lateral cell wall (Fig. 4c to e) (48, 70, 71). A necklace of exposed β-1,3-glucan is present around the septal junction between the mother and daughter cells (Fig. 4b; see Video S3 in the supplemental material). Following cytokinesis, β-1,3-glucan is exposed at the resultant bud scar on the mother cell but not the daughter (Fig. 3), which is consistent with the asymmetric degradation of β-1,3-glucan in the septum via Eng1 synthesized by the daughter cell (67, 68). A consequence of this is that daughter cells are likely to be less visible to innate immune cells than their mother cells (Fig. 8e, compare β-glucan MFI of yeast cells to that of budding yeast cells). However, as macrophages frequently target the bud scar during phagocytosis (Fig. 2), lactate-induced shaving of exposed β-1,3-glucan at bud scars (Fig. 1) is likely to reduce the vulnerability of mother cells to antifungal immune recognition (43).

The ultrastructural nature of the punctate foci of exposed β-1,3-glucan on the lateral cell wall remains obscure. Higher-resolution imaging, for example, via cryo-electron microscopy, is required to resolve the structure of these punctate foci, which, in our experiments, lie below the limit of resolution of the microscope. These foci may arise simply through imperfections in the outer mannan layer of the wall. Certainly, genetic, pharmacological, or immunological perturbation of this mannan layer can lead to β-1,3-glucan exposure (38, 39, 41, 71, 86–89). However, the presence of these punctate structures (Fig. 4f to i) may also be consistent with the egress of particles such as extracellular vesicles through the cell wall. C. albicans is known to secrete extracellular vesicles (90, 91), its cell wall is elastic (92), and particles with diameters of 20 to 60 nm have been shown to traverse the cell wall (93). It is conceivable that in the act of traversing the cell wall, extracellular vesicles drag some β-1,3-glucan from the inner wall, leading to exposure at punctate foci. Lactate exposure reduces the number of these punctate foci (Fig. 1b). This could be mediated by lactate-induced β-1,3-glucan shaving (42, 43), by changes in cell wall elasticity mediated by growth on lactate (92, 94), or by a combination of these mechanisms.

In C. albicans, β-1,3-glucan masking in response to lactate, hypoxia, or iron limitation is dependent on PKA signaling (47, 48). In S. cerevisiae and A. nidulans, the central regulatory protein kinase, PKA, mediates catabolite repression through downstream targets, namely, the histone deacetylase Sin3 and the transcriptional repressor Mig1 (52, 53, 95, 96). Sin3 is a target of PKA phosphorylation in C. albicans (51). Therefore, we reasoned that a PKA-Sin3-Mig1/2 pathway might regulate β-1,3-glucan exposure in C. albicans. While some of the phenotypes we observed for C. albicans *mig1*Δ *mig2*Δ, *sin3*Δ, and *tpk1*Δ *tpk2*Δ cells were consistent with this working hypothesis, others were not. For example, Xog1 levels in the secretome were elevated in *sin3Δ* cells (Fig. 6c), consistent with reduced β-glucan exposure on the *sin3Δ* cell surface (Fig. 7). However, the inactivation of PKA, Sin3, and Mig1/2 had differential effects on *XOG1* and *ENG1* transcript levels (Fig. 6a and b), the levels of these β-1,3-glucanases in the secretome (Fig. 6c), and lactate-induced β-1,3-glucan masking (Fig. 5b). Furthermore, we found no evidence of a physical interaction between Sin3 and Mig1 or Mig2 when we performed proteomics to identify proteins that coimmunoprecipitate with Sin3-FLAG_2_ (Table S2). Therefore, while our data indicate that PKA, Sin3, and Mig1/2 influence Xog1 and Eng1 levels in the secretome and β-1,3-glucan shaving, our data are not consistent with the existence of a simple linear PKA-Sin3-Mig1/2 pathway that mediates these effects. Rather, Sin3 appears to regulate Xog1, while Eng1 seems to be modulated by Mig1/2 (Fig. 6a and b).

Does β-1,3-glucan masking influence C. albicans*-*host interactions *in vivo*? Despite the complexity of these interactions (83), this does appear to be the case. Reduced β-1,3-glucan exposure correlates with enhanced fungal colonization of the gastrointestinal tract (35) and elevated fungal burdens in subdermal abscesses (97). The behaviors of the *tpk1*Δ*tpk2*Δ, *sin3*Δ, and *mig1*Δ *mig2*Δ mutants in *Galleria* confirm that exposure to lactate influences the virulence of C. albicans during disseminated candidiasis (Fig. 9), and this resonates with the observation that lactate promotes systemic candidiasis in mice (77). However, as mentioned above, additional factors such as yeast-hypha morphogenesis, for example, contribute to fungal virulence. Lactate exposure influences additional virulence-related phenotypes, such as stress and drug resistance (76, 77, 92, 98). Furthermore, the regulators PKA (Tpk1/2) and Mig1/2 (and no doubt Sin3) execute a variety of roles in C. albicans. PKA regulates growth, stress resistance, morphogenesis, and candidalysin production (73, 75, 99–101) as well as β-1,3-glucanase levels in the secretome (Fig. 6c) and β-1,3-glucan masking (Fig. 5b). Mig1 and Mig2 influence hyphal development and biofilm formation and regulate carbon metabolism (50), in addition to Eng1 levels (Fig. 6a) and β-1,3-glucan masking (Fig. 5b). Sin3 influences cell morphology (Fig. 7c), as well as Xog1 levels (Fig. 6c) and β-1,3-glucan exposure (Fig. 5b). On top of this, a variety of host-related signals trigger changes in β-1,3-glucan exposure (44–49). Therefore, it is not surprising that the relationship between β-1,3-glucan masking and virulence is complex. Nevertheless, it is striking that exposure to lactate tended to reduce the virulence of *tpk1*Δ*tpk2*Δ, *sin3*Δ, and *mig1*Δ *mig2*Δ cells (Fig. 9). Taken together with the effects of these mutations upon lactate-induced β-1,3-glucan masking (Fig. 5), and the propensity of innate immune cell to attack β-1,3-glucan exposing features (Fig. 2), this is consistent with the idea that β-1,3-glucan masking contributes to the fitness of C. albicans during systemic infection, at least in an invertebrate model.

To summarize, we provide new insight into the nature and regulation of the exposure of a major PAMP in C. albicans and how this influences recognition by innate immune cells and infection.

## MATERIALS AND METHODS

### Strains and culture conditions.

C. albicans strains (see Table S3 in the supplemental material) were inoculated into GYNB (2% glucose plus 0.67% yeast nitrogen base without amino acids, containing the appropriate supplements) (102) from a single fresh colony and grown overnight for 16 h at 30°C and 200 rpm. Cells were then harvested by centrifugation at 3,000 rpm, washed twice with sterilized water, and inoculated into fresh GYNB or GYNB containing 2% (vol/vol) dl-lactate sodium to an optical density at 600 nm (OD_600_) of 0.2. The cells were then grown for 5 h, after which they were fixed with 50 mM thimerosal (Sigma-Aldrich, Dorset, UK).

10.1128/mbio.02605-22.3 TABLE S3 Candida albicans strains used in this study. Download Table S3, PDF file, 0.1 MB. Copyright © 2022 de Assis et al. 2022 de Assis et al. https://creativecommons.org/licenses/by/4.0/ This content is distributed under the terms of the Creative Commons Attribution 4.0 International license.

### Protein extraction and immunoprecipitation.

Protein extractions, protein assays, and immunoprecipitations using anti-FLAG M2 magnetic beads and GFP Selector (Nanotag) were performed as described previously (59).

### Flow cytometry.

β-Glucan exposure on C. albicans cells was quantified by flow cytometry, as described previously (48, 49). Briefly, exponential cells were incubated for 5 h under the conditions specified and then fixed with 50 mM thimerosal (Sigma-Aldrich, Dorset, UK). The cells were then stained with Fc-dectin-1 and anti-human IgG conjugated to Alexa Fluor 488 (Invitrogen), and the fluorescence of 10,000 events was acquired using a Attune NxT flow cytometer or an Amnis ImageStream Mk II imaging flow cytometer. Imaging flow cytometry was performed using an ImageStream at a magnification and extended depth of field of 40×, with 200-μL samples at concentrations of 1 × 10^6^ cells/mL in fluorescence-activated cell sorting (FACS) buffer (phosphate-buffered saline [PBS] containing 1% heat-inactivated fetal bovine serum [FBS] and 0.5 mM EDTA). Median fluorescence intensities (MFIs) were determined using FlowJo v.10 software. Each cytometry plot is representative of at least three independent biological experiments.

For some experiments, C. albicans cells were prestained with calcofluor white (CFW) to differentiate mother cells from daughters. To achieve this, C. albicans SN250 was grown overnight in GYNB at 30°C, washed 3 times in deionized water, inoculated into fresh GYNB at an OD_600_ of 0.2, and grown for 2.5 h at 30°C. These cells were then stained with 20 μg/mL CFW for 5 min and then washed three times with deionized water, and the cells were then grown for additional 2.5 h at 30°C. Control, unstained cells were inoculated into fresh GYNB at an OD_600_ of 0.2, and these cultures were grown for 5 h at 30°C, with collection of unstained samples at 2.5 and 5 h to check the β-glucan levels. Finally, cells (stained and unstained) were harvested, fixed with 50 mM thimerosal, stained with Fc-dectin-1 (above), and analyzed by flow cytometry using an Attune NxT.

### qRT-PCR.

RNA was prepared from cells ground under liquid nitrogen using RNeasy plant mini kits (Qiagen, Manchester, UK), according to the manufacturer’s instructions. cDNA was synthesized from 2 μg RNA using the ImProm-II reverse transcriptase kit (Promega, Southampton, UK), per the manufacturer’s instructions. For qRT-PCR, 1 μL cDNA was used for each SYBR green reaction (SYBR green PCR master mix: Qiagen, Manchester, UK). Transcript levels were normalized using Bio-Rad CFX96 software against the internal *ACT1* and *TAF14* mRNA controls.

### Proteomics.

To examine the secretome, C. albicans cells grown for 5 h in GYNB or GYNB containing 2% lactate were harvested by centrifugation (3,000 rpm for 5 min at 20°C), 100 mL of culture supernatant was concentrated down to 200 μL at 4°C using Amicon Ultra 15- to 10,000-nominal-molecular-weight-limit (NMWL) filters (Sigma-Aldrich, Gillingham, UK), and the samples were frozen using liquid nitrogen. The samples were then freeze-dried before trypsin digestion.

To identify Sin3-FLAG_2_ interacting proteins, C. albicans Ca1428 cells (Table S1) were grown in GYNB or GYNB containing lactate for 5 h, protein extracts were prepared, and immunoprecipitations were performed as described above. These immunoprecipitated proteins were then subjected to trypsin digestion.

Trypsin digestions were performed by resuspending samples in 93 μL of 50 mM ammonium bicarbonate, 1 μL of 0.5 M dithiothreitol (DTT) was added, and the samples were incubated at 56°C for 20 min. Next, 2.7 μL of fresh 0.55 M iodoacetamide was added, and the samples were incubated at room temperature for 15 min in the dark. After these reduction and alkylation steps, 1 μL of ProteaseMAX surfactant (V2071; Promega, Southampton, UK) and 1 μL of trypsin gold, mass spectrometry grade (Promega V5280), were added, and the samples were mixed and incubated overnight at 37°C. Reactions were terminated by adding 1 μL of trifluoroacetic acid (liquid chromatography-mass spectrometry [LC-MS] grade), and incubated for an additional 5 min. LC-tandem MS (LC-MS/MS) scans and analyses were performed with three independent replicates using the parameters described previously (103).

### Fluorescence microscopy.

C. albicans cells were grown in GYNB or GYNB containing 2% lactate for 5 h at 30°C (as above), fixed overnight with 50 mM thimerosal, washed twice with deionized water, and stained with Fc-dectin-1 and IgG-AF488 (β-1,3-glucan) and ConA-AF647 (mannan). Fixed, unstained cells were used as controls. Slides were prepared by pipetting 300 μL of 2% (wt/vol) agar onto a glass slide, and a second slide was then placed on top to produce a cushion that was approximately 1 mm thick. Once the agar had set (20 min), 3 μL of cell suspension was pipetted onto the agar cushion, and a Zeiss 1.5 thickness high-performance coverslip was place on top (18 by 18 mm; 0.170 ± 0.005 mm). Cells were then observed and imaged using a Zeiss LSM880 confocal system using Airyscan high-resolution modes (Zeiss Zen [blue edition] 2.3). The system was equipped with an alpha Plan-Apochromat 100×/1.46 oil DIC objective. Nyquist sampling in xyz dimensions was used. The sample was excited with a 561-nm (20-mW) and a 488-nm (25-mW) laser, with power set to 5% for both tracks. The main beam splitter was a Zeiss dual pass 488/561 filter, and the other Airyscan path filters were set to plate. All images were then Airyscan processed (Zeiss Zen [blue edition] 2.3) using an automatic Wiener filter setting. Images were subsequently linearly unmixed to adjust for cross talk using automatic two-component extraction (Zeiss Zen [blue edition] 2.3). Contrast and brightness were adjusted, and parameters were set to similar values across all treatments. Scale bars were applied, 2D and 3D projections were generated, and images were exported as 16-bit tiff files or high-resolution Windows media files.

For quantitative fluorescence microscopy of β-1,3-glucan-exposing features, C. albicans SN250 cells were grown in GYNB, fixed with 50 mM thimerosal, and stained with 1.5 μg/mL Fc-dectin-1 plus anti-human IgG conjugated to Alexa Fluor 488. Using a Zeiss Axioplan 2 microscope, cells were analyzed by phase DIC and fluorescence microscopy. The images were recorded using Openlab v.4.04 (Improvision, Coventry, UK) with a Hamamatsu C4742-95 digital camera (Hamamatsu Photonics, Hamamatsu, Japan). The volume and intensity of β-1,3-glucan-exposing features were quantified from cells fixed with thimerosal and stained with Fc-dectin-1. Cells were suspended in ProLong diamond antifade mountant (Invitrogen). 3D images were captured using a Nikon Eclipse Ti UltraVIEW VoX spinning-disk microscope. Volocity software was used to identify objects in the 488 channel and to measure their volume and mean fluorescence intensity. Septal junctions, bud scars, and parts thereof were relatively large (>2 μm^3^). Punctate foci ranged in volume but were smaller (<2 μm^3^).

### Macrophage attachment and engulfment.

Bone marrow-derived macrophages (BMDMs) were prepared from bone marrow from femurs and tibias of 12-week-old male C57BL/6 mice and differentiated for 7 days, as described previously (104). BMDMs were mixed with C. albicans SC5314 cells (1:3 macrophage-to-yeast cell ratio) that had been grown in the presence or absence of lactate. The BMDM-*Candida* interactions were imaged in DIC using a Nikon Eclipse Ti UltraVIEW VoX spinning-disk microscope at 5-s intervals for 60 min, and the videos were analyzed using Volocity software. The positions at which BMDMs first attached to *Candida* cells and the time from this first attachment to the full engulfment of the *Candida* cargo were monitored. The data represent events from three independent experiments (videos) per condition: *n* = 48 for glucose; *n* = 52 for glucose plus lactate.

### Galleria mellonella infections.

Galleria mellonella infections were performed as described previously (78). The wax moth Galleria mellonella was obtained from UK Waxworms, Ltd. (Sheffield, UK). C. albicans strains were grown in GYNB with or without lactate for 5 h at 37°C, and the cells were washed and resuspended in PBS. Upon arrival, the larvae were kept in wood shavings at 12°C for up to 2 weeks before the experiments. Initially, to compare killing rates for wild-type and deletion strains, groups of 15 larvae weighing 200 to 300 mg were inoculated with 1 × 10^8^, 5 × 10^7^, 5 × 10^6^, and 4 × 10^5^ cells/mL of the wild-type strain. Based on these comparisons, a dose of 2.5 × 10^5^ cells was used in subsequent experiments. Each larva was injected with 50 μL of fungal cell suspension through the second proleg, using a 0.5-mL BD Micro-Fine insulin syringe, and noninjected and PBS controls were included. The larvae were then incubated at 37°C in the dark, and survival was quantified for 10 days postinfection.

### Data analysis and statistics.

C. albicans mutants were compared to the appropriate isogenic parental strain. Flow cytometry data were analyzed using FlowJo 10.7.2 software for MacBook, and ImageStream analyses were performed using IDEAS 6.3 software. High-resolution confocal microscopy images were analyzed using ZEISS Zen lite, blue and black, version 3.4, and processed images were smoothed using IMARIS software version 9.7. Figures were prepared using Adobe Illustrator 2021 and Adobe Photoshop 2021 for MacBook. Data are expressed as means ± standard deviation of results from at least three independent experiments, and statistical analyses were performed using analysis of variance (ANOVA) with Tukey’s multiple-comparison test or log rank (Mantel-Cox) test for the survival curves in Prism GraphPad 9.0 (*, *P* < 0.05; **, *P* < 0.01; ***, *P* < 0.001).
