# Supplementary figures and images for: Nature of β-1,3-Glucan-Exposing Features on Candida albicans Cell Wall and Their Modulation
Source: mBio. 2022 Oct 11;13(6):e02605-22. doi: 10.1128/mbio.02605-22 (PMC9765427; doi:10.1128/mbio.02605-22)

a

## Growth after 5 h

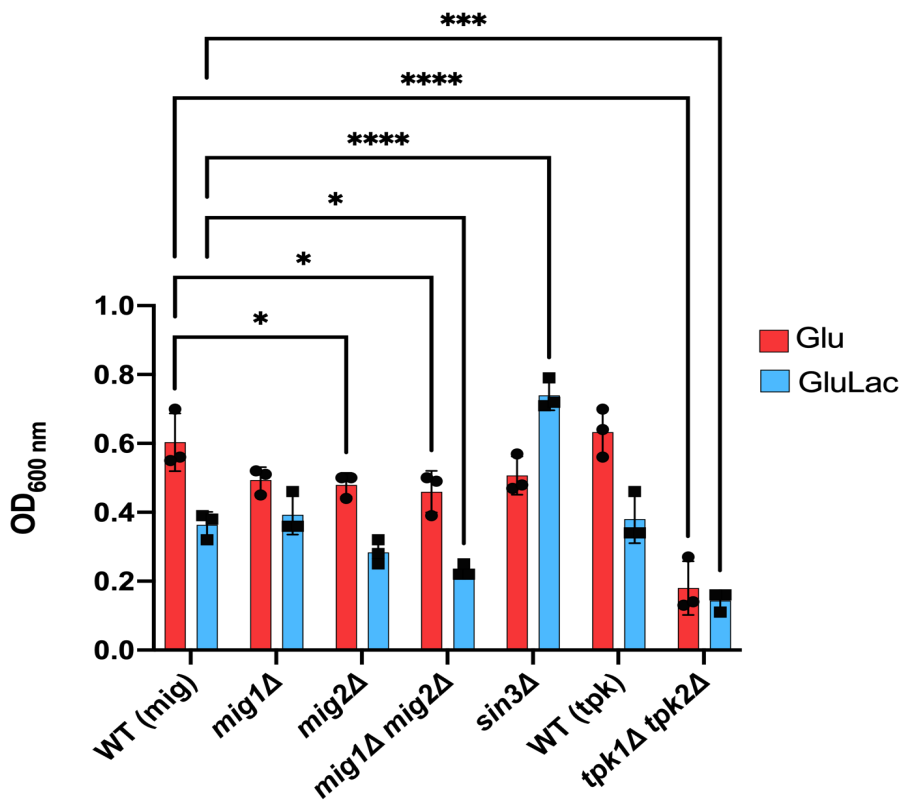

b

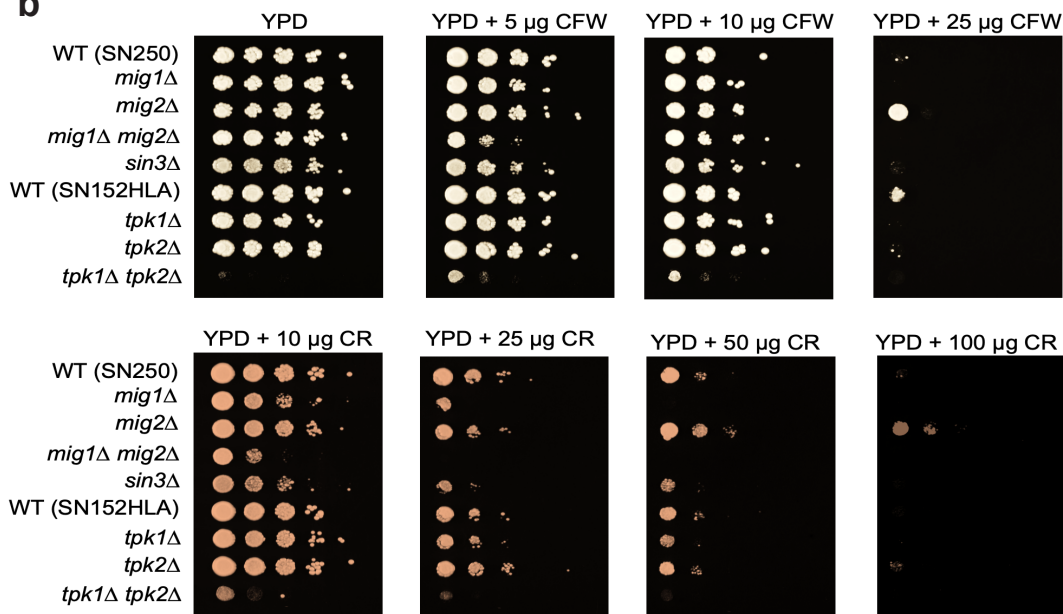

Supplement: FIG S1 [file mbio.02605-22-s0007.pdf]
