# Supplementary material for: Nature of β-1,3-Glucan-Exposing Features on Candida albicans Cell Wall and Their Modulation
Source: mBio. 2022 Oct 11;13(6):e02605-22. doi: 10.1128/mbio.02605-22 (PMC9765427; doi:10.1128/mbio.02605-22)
Supplement: TABLE S3 [file mbio.02605-22-s0010.pdf]

Table S3. Primers used for RT-qPCR

Target	*F primer*	*R primer*	*Reference*
Human IFN-β	CAGCAATTTTCAGTGTCAGAAGC	TCATCCTGTCCTTGAGGCAGT	(1)
Human ISG15	GCGAGATCACCCAGAAGATT	GCCCTTGTTATTCCTCACCA	(2)
Human IFIT1	GCGCTGGGTATGCGATCTC	CAGCCTGCCTTAGGGGAAG	(3)
KSHV ORF37	TGACACCCTTGGGTTAACAGT	TCTCGAACCTTGGCGTGCTTTAGA	(4)

References

1. Jacobs SR, Gregory SM, West JA, Wollish AC, Bennett CL, Blackbourn DJ, Heise MT, Damania B. 2013. The Viral Interferon Regulatory Factors of Kaposi's Sarcoma-Associated Herpesvirus Differ in Their Inhibition of Interferon Activation Mediated by Toll-Like Receptor 3. J Virol 87:798–806.

2. Yoo Y-S, Park Y-Y, Kim J-H, Cho H, Kim S-H, Lee H-S, Kim T-H, Sun Kim Y, Lee Y, Kim C-J, Jung JU, Lee J-S, Cho H. 2015. The mitochondrial ubiquitin ligase MARCH5 resolves MAVS aggregates during antiviral signalling. Nat Commun 6:7910.

3. Gardinassi LG, Garcia GR, Costa CHN, Silva VC, Santos IKF de M. 2016. Blood Transcriptional Profiling Reveals Immunological Signatures of Distinct States of Infection of Humans with Leishmania infantum. PLoS Negl Trop Dis 10:e0005123.

4. Rossetto CC, Pari G. 2012. KSHV PAN RNA Associates with Demethylases UTX and JMJD3 to Activate Lytic Replication through a Physical Interaction with the Virus Genome. PLOS Pathog 8:e1002680.
